# Mycotoxins in Poultry Feed and Feed Ingredients from Sub-Saharan Africa and Their Impact on the Production of Broiler and Layer Chickens: A Review

**DOI:** 10.3390/toxins13090633

**Published:** 2021-09-08

**Authors:** Phillis E. Ochieng, Marie-Louise Scippo, David C. Kemboi, Siska Croubels, Sheila Okoth, Erastus K. Kang’ethe, Barbara Doupovec, James K. Gathumbi, Johanna F. Lindahl, Gunther Antonissen

**Affiliations:** 1 Laboratory of Food Analysis, FARAH-Veterinary Public Health, University of Liège, Avenue de Cureghem 10, 4000 Liège, Belgium; peochieng@uliege.be (P.E.O.); mlscippo@ulg.ac.be (M.-L.S.); 2 Department of Pharmacology, Toxicology and Biochemistry, Faculty of Veterinary Medicine, Ghent University, Salisburylaan 133, 9820 Merelbeke, Belgium; David.Kemboi@UGent.be (D.C.K.); Siska.Croubels@UGent.be (S.C.); 3 Department of Pathology, Microbiology and Parasitology, Faculty of Veterinary Medicine, University of Nairobi, P.O. Box 29053, Nairobi 00100, Kenya; jkgathumbi@uonbi.ac.ke; 4 Department of Animal Science, Chuka University, P.O. Box 109-00625, Chuka 00625, Kenya; 5 School of Biological Sciences, University of Nairobi, P.O. Box 30197, Nairobi 00100, Kenya; sheilaokoth@uonbi.ac.ke; 6 Independent Researcher, P.O. Box 34405, Nairobi 00100, Kenya; mburiajudith@gmail.com; 7 BIOMIN Research Center, Technopark 1, 3430 Tulln, Austria; barbara.doupovec@dsm.com; 8 Department of Biosciences, International Livestock Research Institute (ILRI), P.O. Box 30709, Nairobi 00100, Kenya; J.Lindahl@cgiar.org; 9 Department of Medical Biochemistry and Microbiology, Uppsala University, P.O. Box 582, 751 23 Uppsala, Sweden; 10 Department of Clinical Sciences, Swedish University of Agricultural Sciences, P.O Box 7054, 750 07 Uppsala, Sweden; 11 Department of Pathology, Bacteriology and Avian Diseases, Faculty of Veterinary Medicine, Ghent University, Salisburylaan 133, 9820 Merelbeke, Belgium

**Keywords:** aflatoxins, co-occurrence, eggs, fumonisins, mycotoxicosis, poultry feeds

## Abstract

The poultry industry in sub-Saharan Africa (SSA) is faced with feed insecurity, associated with high cost of feeds, and feed safety, associated with locally produced feeds often contaminated with mycotoxins. Mycotoxins, including aflatoxins (AFs), fumonisins (FBs), trichothecenes, and zearalenone (ZEN), are common contaminants of poultry feeds and feed ingredients from SSA. These mycotoxins cause deleterious effects on the health and productivity of chickens and can also be present in poultry food products, thereby posing a health hazard to human consumers of these products. This review summarizes studies of major mycotoxins in poultry feeds, feed ingredients, and poultry food products from SSA as well as aflatoxicosis outbreaks. Additionally reviewed are the worldwide regulation of mycotoxins in poultry feeds, the impact of major mycotoxins in the production of chickens, and the postharvest use of mycotoxin detoxifiers. In most studies, AFs are most commonly quantified, and levels above the European Union regulatory limits of 20 μg/kg are reported. Trichothecenes, FBs, ZEN, and OTA are also reported but are less frequently analyzed. Co-occurrences of mycotoxins, especially AFs and FBs, are reported in some studies. The effects of AFs on chickens’ health and productivity, carryover to their products, as well as use of mycotoxin binders are reported in few studies conducted in SSA. More research should therefore be conducted in SSA to evaluate occurrences, toxicological effects, and mitigation strategies to prevent the toxic effects of mycotoxins.

## 1. Introduction

The poultry industry in sub-Saharan Africa (SSA) is an essential subsector of agriculture, providing food, employment, and other economic resources for the region. As an example, over 80% of households in Ethiopia and Swaziland are reported to practice poultry farming at small-scale levels, as it requires less space compared with other livestock, such as dairy and pigs [1,2]. Poultry and fish proteins contribute over 60% of the human protein intake in SSA, and demand for animal proteins is projected to increase due to the rapid growth of the human population, which is projected to be 2.2 billion by 2050 [3]. Furthermore, urbanization and increases in gross domestic product (GDP) also contribute to the demand for animal source foods [4]. Poultry production in Southern and Eastern African countries have gradually grown over the past decades to commercial poultry value chains that include feed suppliers, hatcheries, housing, and slaughtering equipment, as well as veterinary services [5]. This growth in production systems will inevitably become more intensive and thereby increase the demand for high-quality poultry feeds.

Poultry feeds in SSA, similar to other parts of the world, consist of animal protein sources, such as fish meal, meat, and bone meal, whereas major plant protein sources include soybean meal, cotton seed, sunflower seed cake, and peanut products, with maize serving as the main source of energy [6]. Fish meal and soybean meal are the conventional protein sources and currently becoming scarce and expensive, thereby making poultry feeds costly and accounting for approximately 60% to 80% of the total production cost [7]. In addition, feed ingredients have been shown to be prone to contamination by mycotoxins, resulting in contamination of final poultry feed commodities [6,8].

Mycotoxins are secondary metabolites of fungi mainly belonging to the genera *Aspergillus*, *Alternaria*, *Fusarium*, *Cladosporium*, *Claviceps*,and *Penicillium* [9]. More than 400 mycotoxins have been reported in food and feed. The most frequently detected, and of concern globally, are aflatoxin B1 (AFB1), aflatoxin B2 (AFB2), aflatoxin G1 (AFG1), and aflatoxin G2 (AFG2); fumonisins (FBs); trichothecenes (for example, deoxynivalenol (DON) and T-2 toxin (T-2)); and ochratoxin A (OTA) [10]. These toxins are reported to cause economic losses, diseases, and even the death of humans and animals. In SSA, the risk of food and feed contamination with mycotoxins has been reported to be considerably high as adequate control and good storage are difficult to achieve [11,12]. Climatic conditions in most regions in SSA are characterized by high relative humidity, high temperatures, and little aeration [13]. These conditions make crops more liable to mycotoxin contaminations as they accelerate growth and mycotoxin biosynthesis by toxigenic fungi. Although the focus of mycotoxin risk assessment and management in developing countries is on food safety, given the direct links between feed safety, food production, and the safety of animal food products, it is essential that feed production and manufacturing procedures meet safety requirements. This review summarizes occurrences of mycotoxins in poultry feed and feed ingredients from SSA over the last 23 years (between the years 1998 and 2021). Worldwide regulations of mycotoxins in poultry feeds and the impact of mycotoxins on layer and broiler chickens’ production as well as the use of mycotoxin binders as postharvest mycotoxin mitigation strategies are also reviewed.

## 2. Worldwide Regulation of Mycotoxins in Poultry Feeds

In Africa, only 6 countries out of 54 and 1 region (East African Community, (EAC)) had regulatory limits for AFs in poultry feeds (Table 1) at the time of their review. South Africa was the only country with guidance values for OTA, FBs, and DON in poultry feeds, in addition to regulatory limits for AFs [6]. The East African Community, a regional intergovernmental organization, has set the maximum limit for total AFs at 50 μg/kg and AFB1 at 20 μg/kg for adult poultry feeds [14]. Most African regional and national mycotoxin regulatory limits are established and enforced due to trade and the desire to comply with export regulations [14,15]. The European Union (EU) has regulations and guidance values for mycotoxins in feeds for different animal species and has established regulatory limits for AFB1 and guidance values for total FBs, DON, ZEN, and OTA in poultry feeds (Table 1) [16]. On the other hand, the Canadian Food Inspection Agency (CFIA) set guidance values for both OTA and T-2 in poultry feed at levels higher than those recommended by the EU [17]. Similarly, the United States of America has set higher guidance values for DON and FBs in poultry feeds as compared with those set by the EU [18].

## 3. Occurrence of Major Mycotoxins in Poultry Feed and Feed Ingredients from SSA

Few studies have been conducted to determine mycotoxins in poultry feeds and poultry feed ingredients, with AFs being the most determined mycotoxins (Table 2).

### 3.1. Aflatoxins

Aflatoxins occurrences of between 64% and 100% have been reported in poultry feeds [4,13,31,33]. Levels above the EU regulatory limit of 20 μg/kg are reported mainly in countries in the tropical regions, including Nigeria [8,31], Ghana [4], Kenya [10], and Uganda [11]. This could be attributed to warm and humid tropical conditions coupled with poor agricultural practices that favor AFs production in these regions. On the other side, low occurrences and low levels (range: 0.3 to 0.7 μg/kg) of AFs were reported in poultry feeds from South Africa [6,33], possibly due to variations in climatic conditions that affect the ecological niche of parent fungi and AFs accumulation. In feed ingredients, high occurrences and levels of AFs are mainly reported in oilseeds, such as peanut and sunflower, as well as their products. Over 60% of peanut from Benin [28], Cameroon [13], Ethiopia [22,35], and Nigeria [8,32] were contaminated with AFs, and levels up to 11,900 μg/kg were reported. Similarly, sunflower cakes and seeds from Tanzania [26] and Kenya [34] had high AFs levels (max: 662.7 μg/kg). Maize and maize products are also frequently contaminated with AFs. All maize samples from Malawi [24], Uganda [11], and Ethiopia [23] were contaminated with AFs at maximum levels of 150 μg/kg. Lower incidences of below 50% were reported in maize samples from other SSA countries, although with levels as high as 567 μg/kg [8,27,29].

Besides AFB1, other AFs, including AFB2, AFG1, AFG2, and aflatoxin M1 (AFM1), have also been reported in poultry feeds and feed ingredients [8,10,33,34]. More than half of poultry feeds from Nigeria [8,31] and South Africa [33] were contaminated with AFB2 at levels up to 188 μg/kg. Moreover, over 90% of peanut cake from Nigeria had AFB2 at a maximum level of 895 μg/kg [8]. In contrast, less than half of poultry feed and feed ingredients from Kenya [10] and other 13 African countries [25] were contaminated with AFB2. Aflatoxin G1 is the second most prevalent AFs in SSA besides AFB1 and has been reported in over 50% of poultry feeds [8,10,33]. The highest AFG1 levels of 235 μg/kg (mean: 45 μg/kg) were reported in poultry feeds from Nigeria [31]. In feed ingredients, AFG1 occurred in over 80% of peanut meal from Nigeria at levels up to 568 μg/kg [8,32]. Furthermore, levels up to 725 μg/kg were observed in maize from the same country [8]. Aflatoxin G2 has also been reported in over half of poultry feeds from South Africa [33] and feed and feed ingredients from Nigeria [34]. In contrast, less than half of poultry feeds from Kenya [10] and Nigeria [8,31] had detectable AFG2. In feed ingredients, the highest occurrences (55%) and levels (68 μg/kg) of AFG2 were reported in peanut cake from Nigeria [8]. The presence of AFM1 in feeds and feed ingredients from SSA has been associated with the production of traces of AFM1 by most strains of aflatoxigenic *Aspergilli* [31]. Low occurrences of less than half of poultry feed samples from Kenya [10] and Nigeria [8,31] were reported at maximum levels of 41 μg/kg. Interestingly, high occurrences of over 60% and levels up to 254 μg/kg were reported in peanut cake samples from Nigeria [8,32].

### 3.2. Fumonisins

Fumonisins are the second most common mycotoxins, and high occurrences of over 80% were reported in poultry feeds from South Africa [6,33], Kenya [10], Nigeria [8,31], and Ghana [4], although at levels below the EU guidance value of 20,000 μg/kg for FBs in poultry feeds. Low occurrences of below 50% (range of means: 572 to 1210 μg/kg) were however reported in poultry feeds from Botswana [20] and Rwanda [15]. The low occurrences and levels in the latter studies could be attributed to detection methods and sampling periods. In feed ingredients, over 70% of samples from Kenya [10], Nigeria [8], and Ethiopia [23] were contaminated, with FBs at maximum levels of 11,831 μg/kg. However, less than 40% of maize samples from Ethiopia [23,29] and Tanzania [27] had detectable FBs but at levels up to 62,000 μg/kg. Unlike maize, peanuts and peanut products are not frequently contaminated with FBs, and less than half of samples from Nigeria [8,32] and Benin [28] contained FBs (max: 910 μg/kg).

### 3.3. Deoxynivalenol

High occurrences of DON have also been reported in poultry feeds, although usually at levels below both South African and EU guidance values [6,8,10,31,33]. Almost all poultry feed samples from South Africa [6,33] and Kenya [10] were contaminated with DON at maximum levels of 1980 μg/kg. However, DON was not a major contaminant of poultry feeds from Nigeria [8,31]. The low occurrences sometimes observed in samples from countries along the tropical regions can be due to DON most commonly being produced in temperate regions by pathogenic plant fungi, including *Fusarium graminearum* and *F. culmorum* [36]. Poultry feed ingredients such as maize and wheat are however frequently contaminated with DON [10,25,34], and levels up to 11,022 μg/kg (mean: 943 μg/kg) were detected in wheat from South Africa [34]. Furthermore, DON was present in over half of feed ingredients from Kenya in the range of 22 to 996 μg/kg [10]. However, Worku et al. [23] reported DON in only 7% of maize samples from Ethiopia at concentration ranges of 270 to 1980 μg/kg.

### 3.4. Zearalenone

Similar to DON and FBs, ZEN is reported to be a frequent contaminant of poultry feeds and was detected in over 50% of poultry feeds from Kenya [10], South Africa [6,33], and Nigeria [8,31]. Levels above the EU guidance value of 250 μg/kg were reported in poultry feeds from Kenya [10] and South Africa [6,33]. Ezekiel et al. [31], however, reported ZEN in only 22% of poultry feeds from Nigeria at mean concentrations of 45 μg/kg. Poultry feed ingredients, especially maize, are also reported to be frequently contaminated with ZEN [10,25,29], and levels up to 6276 μg/kg were observed in maize from South Africa [25], while a small study found levels up to 165,000 μg/kg [37]. However, low occurrences of ZEN in less than half of maize from other African countries [20,25] and peanut cake samples from Nigeria [8] were reported.

### 3.5. T-2 and HT-2 Toxins

There is limited information on occurrences of T-2 and HT-2 toxins in feed and feed ingredients from SSA, and the few studies indicate that they are not major contaminants in this region. Occurrences of both T-2 and HT-2 toxins were reported in 4% of poultry feeds from Kenya at mean levels of 13.8 and 5.2 μg/kg, respectively [10]. However, Mokubedi et al. [33] reported occurrences of both T-2 and HT-2 toxins in all tested poultry feeds from South Africa at mean concentrations of 3.1 and 1.9 μg/kg, respectively. In feed ingredients, T-2 toxin occurred in less than 5% of maize from different countries in Africa at maximum levels of 80 μg/kg [25].

### 3.6. Ochratoxin A

Similar to T-2 and HT-2 toxins, OTA is not an important contaminant of poultry feeds in the region, and low occurrences of less than 34% (range of means: 4.8 to 10.0 μg/kg) were reported in poultry feeds from Nigeria [8,31] and Kenya [10]. In the latter studies, low levels of OTA were found together with high occurrences and levels of AFs, which could be attributed to OTA being produced by the same *Aspergillus* spp. fungi that produce AFs. In feed ingredients, low occurrences and levels were further reported in samples from Kenya [10], Nigeria [8,34], and South Africa [23]. Gruber-Dorninger et al. [25], however, reported OTA at levels up to 694 μg/kg in maize samples from different African countries. Similar to maize, peanut and peanut products are reported to be susceptible to infestation by *Aspergillus* spp. fungi and thus prone to contaminations by OTA. Over half of peanut cake samples from Nigeria had OTA at maximum levels of 127 μg/kg [8]. In contrary results, OTA was detected in less than 33% of peanut cake samples from Benin [28] and Nigeria [32].

### 3.7. Co-Occurrence of Mycotoxins

Co-occurrence of mycotoxin in poultry feeds has been observed in field conditions since the different feed ingredients are colonized by more than one fungus and because most fungi can produce several mycotoxins simultaneously [12]. Contamination of poultry feeds and feed ingredients by more than one mycotoxin is reported in various studies [4,8,10,23,25,31]; however, there is likely underestimation since not all known mycotoxins are tested, and in some studies, the methods employed are not capable of detecting all mycotoxins of interest. Aflatoxins and FBs are the most frequently reported co-occuring mycotoxin combination and were recently found to co-occur in all tested poultry feeds from Ghana [4]. This co-occurrence was also the most common in poultry feeds and feed ingredients in other studies [8,10,15,23,25]. Besides co-occurring with FBs, AFs were recently reported to co-occur with ZEN in all feed and feed ingredients from Kenya, whereas AFs and DON co-occurred in over 80% of the samples [10]. Furthermore, co-occurrences of AFs with FBs, ZEN, and DON were reported in over half of poultry feeds from South Africa [33]. In the latter study, FBs, ZEN, and DON co-occurred in 42% of the samples. Njobeh et al. [6] also reported co-occurrence of FBs, DON, and ZEN in over 60% of feeds from South Africa, whereas FBs, DON, ZEN, and AFs co-occurred in 26% of the samples. Co-occurrence of five mycotoxins, including FBs, DON, ZEN, AFs, and OTA, in about 0.1% of the samples was also reported in the latter study. Interestingly, Olopade et al. [38] recently reported that DON and ZEN did not co-occur in maize, sorghum, and millet samples from Nigeria. Co-occurrences of mycotoxins can lead to toxicological interactions, thereby making some mycotoxins harmful even at low levels due to synergistic or additive effects [16].

## 4. Mycotoxins in Poultry Food Products from Surveys Conducted in SSA

Occurrences of mycotoxin residues in biological samples, especially animal source foods, were used to evaluate mycotoxin exposure in animals and assess the risk to human consumers [39]. Although various studies indicate that chicken eggs and meat are only minor contributors to human dietary mycotoxin exposure [40,41], chronic exposure to these low levels could have a negative impact on the health of human consumers [42,43]. Iqbal et al. [44] reported the highest total AFs concentration of 8 μg/kg in livers of chickens compared with other organs, because the liver is the primary target organ of AFs. Using ELISA methods, AFB1 was detected in 39% of liver samples (mean level: 1.7 μg/kg) and about 14% of gizzard samples (mean level: 1.1 μg/kg) in Mozambique [42]. Mycotoxin residues have also been detected in chicken eggs, although low transmission rates of below 1% from feed to eggs are often reported [40,45]. Using HPLC methods, Tchana et al. [46] reported total AFs in about 45% of egg samples from Cameroon at levels up to 7.6 μg/kg (mean: 0.8 μg/kg).

## 5. Aflatoxicosis Outbreaks in Poultry in Africa

Aflatoxicosis outbreaks linked to consumption of contaminated feed and feed ingredients have been reported to cause death of a large number of poultry in Kenya and Morocco [47]. Local or imported feed and feed ingredients (especially maize and groundnuts) have been associated with three aflatoxicosis outbreaks in different parts of Kenya [47,48]. During the aflatoxicosis outbreaks, large numbers of poultry were affected, with death being the major effect reported. In Morocco, consumption of feed contaminated with AFs up to levels of 5625 μg/kg resulted in aflatoxicosis outbreak that affected a large number of poultry [49]. In the latter study, death of poultry was again the reported effect.

## 6. Toxicological Impact of Major Mycotoxins on Experimental Broilers and Layer Chickens

Consumption of mycotoxin-contaminated feeds has been reported to cause poor health and performance of chickens as well as mortalities (Table 3); however, there are limited data in the SSA context. Apart from a few studies, mostly conducted in Nigeria [50,51], the impacts are reported from trials conducted in other countries. Effects of a mycotoxin on an animal depend on the mycotoxin type, level and duration of exposure, age, and specie of the animal. Acute toxicity is caused by intake of high doses of mycotoxins and is characterized by death and well-described clinical signs [37]. Aflatoxins are reported to cause reduced bird performance, lower immunity, organ damage, and reduced egg production [52,53,54,55]. On the other hand, toxicity due to FBs has been linked to disruption of the biosynthesis of sphingosine (So) and sphinganine (Sa) due to structural similarity between these sphingolipids and FBs backbone [56,57,58]. Furthermore, FBs have been reported to cause nephrotoxicity, diarrhea, reduced body weight gain, and organ damage in chickens [59,60,61]. Trichothecenes, including DON, are mainly reported to exhibit toxicity through inhibition of protein synthesis and bonding to sulfhydryl groups at the subcellular, cellular, and organic system levels [16,45,58]. Several studies have indicated that animals, including poultry, are naturally exposed to high doses of trichothecenes compared with humans, with reduced weight and immunosuppression being observed in chronic exposures, whereas vomiting, nausea, irritation, and lesions are reported in acute exposures [60,62,63]. Zearalenone is reported to have estrogen-mimicking effects, and its toxicity has been associated with damaged reproductive organs [64,65]. Toxicity due to OTA has been shown to be through generation of DNA adducts that cause impairment of protein synthesis, increased oxidative stress, and inhibition of mitochondrial function [66,67]. Subacute levels of OTA are reported to be nephrotoxic, immunosuppressive, and teratogenic in animals [67,68].

### 6.1. Immunosuppression, Susceptibility to Diseases, and Mortality

The immunosuppressive effects of mycotoxins have been shown to cause reduced disease resistance, reduced drug efficacy, and vaccine failures, making the animal more susceptible to diseases and increasing mortalities [19,55,60,88]. Farmers, therefore, incur hidden costs related to the treatment of diseased animals and economic losses due to mortalities. In Nigeria, the highest mortality was reported in broilers fed 500 μg AFB1/kg feed [51]. The levels of AFB1 used in the latter study are practically relevant since levels up to 1067 μg/kg were reported in poultry feeds from the same country [8], implying that the poultry sector is at risk. Other studies on the effects on poultry in SSA are lacking, but there are data from trials worldwide that are also relevant for the SSA context. Naseem et al. [89] reported the highest mortality in young layers fed 200 μg AFB1/kg feed and challenged with fowl adenovirus 4. Similarly in another study, the highest mortality was observed in broiler chickens fed AFB1 (750 or 1500 μg/kg feed) and challenged with *Clostridium perfringens* pathogen [90]. For OTA, the highest mortality was reported in broiler chickens fed 1000 μg OTA/kg feed and challenged with coccidia [91], and mortality of about 13% was observed in young broilers fed 800 μg OTA/kg feed [84]. Mycotoxins, even at subclinical levels, lead to immunological and metabolic disturbances, thereby enhancing diseases in chickens. Enhanced coccidiosis was observed in broilers fed 1000 μg OTA/kg diet and challenged with coccidia [91]. Moreover, mycotoxins such as DON and FBs are poorly absorbed and remain in the gastrointestinal tract, impairing highly dividing intestinal cells and providing growth substrates for the colonization of the digestive system by pathogens [60,61]. Increased necrotic enteritis was reported in chickens fed FBs-contaminated diets and challenged with *C. perfringens* [60]. Additionally, feeding subclinical doses of *Fusarium* mycotoxins, including FBs, DON, 15-acetyl DON, and ZEN, enhanced coccidiosis or retarded recovery in coccidia-challenged broiler chickens [61,92].

Reduced immune responses characterized by reduced antibody titers against vaccines, such as infectious bronchitis vaccine (IBV) and Newcastle disease vaccine (NDV), have been associated with immunosuppressive effects of some mycotoxins [71,93]. Immunotoxic doses of mycotoxins have been observed to be less than the doses required to elicit a reduction in bird performance, and levels such as those reported in poultry feeds from SSA can be immunotoxic. In a feeding trial, immunosuppressive effects of AFB1 (50 or 200 μg/kg feed) or FB1 (50,000 or 200,000 μg/kg), individually or combined, were reported to reduce antibody titers against NDV in broiler chickens [71]. Additionally, dose-dependent decreases in antibody titers against IBV in broiler chickens fed low to moderate DON levels (1680 to 12,209 μg/kg feed) were associated with immunosuppression [94]. Kamalavenkatesh et al. [93] also demonstrated that reduced hemagglutination inhibition titers to NDV in broilers fed CPA (10,000 μg/kg feed) or T-2 (1000 μg/kg feed), alone or in combination, were a result of the immunosuppressive effects of the two toxins. Feeding chickens OTA at levels of 150 to 1000 μg/kg feed resulted in immunosuppressive effects characterized by reduced antibody titers against sheep red blood cells [67,84]. Additionally, decreases in leukocyte counts are also indicative of damaged lymphoid tissues and impaired immune function. Feeding OTA at levels of 20 or 50 μg/kg body weight per day was reported to reduce leukocyte counts in young broiler chickens [82], and Li et al. [95] observed decreased lymphocytes in chickens fed 200,000 μg FB1/kg feed. The immunosuppressive effects of CPA (10,000 μg/kg feed) or T-2 (1000 μg/kg feed), alone or in combination, were reported to cause decreases in lymphocytes of broiler chickens [93]. Chen et al. [87] demonstrated that T-2 toxin (500 to 2000 μg/kg feed) impaired splenic immune function and was manifested through increased apoptotic splenocytes and reduced CD4+/CD8+ T cells. Aflatoxicosis has also been linked to Vitamin A deficiencies in poultry causing reduced immune responses and enhanced susceptibility to diseases, and in a feeding trial, hepatic vitamin A levels were reduced in young broiler chickens fed high AF levels (5000 μg/kg feed) [76].

### 6.2. Impaired Growth

Retarded growth resulting from mycotoxin-contaminated feeds results in poultry not attaining the required weights at the required time and thus economic losses as farmers use more feed and time to attain the required weights [88]. In Nigeria, broilers fed diets contaminated with AFB1 at levels of 34 μg/kg [50] or 500 μg/kg [51] had reduced feed intake and body weight (BW) gain compared with birds fed AFs-free diets. Decreased BW and BW gain were reported in broilers fed AFs at levels of 50 to 200 μg/kg [64,71,96]. The levels of AFs used in the previous studies have been reported in poultry feeds and feed ingredients from SSA, implying that AFs pose a concern to poultry production in the region. High AFs levels of between 2000 and 5000 μg/kg feed caused reduced growth rates in broiler chickens [69,72,73,75,97], with a decrease of about 35% being observed in one of the studies [98]. Diets contaminated with FBs are also reported to cause poor performance through feed refusal and diarrhea, and in experimental studies, FBs at levels of 10,000 μg/kg feed [99] or 200,000 μg/kg feed [71] resulted in decreased broiler chicken BW and BW gain and increased feed conversion ratio (FCR). Moreover, toxicity due to DON has been associated with reduced feed intake and hence poor growth rate. Feeding DON-contaminated diets at low to moderate levels (1680 to 12,209 μg/kg feed) resulted in decreased BW and BW gain of broiler chickens [62,94,100]. While these studies have not been conducted in SSA, similar levels of DON have been reported in poultry feed and feed ingredients from SSA, indicating that DON can have a negative impact on poultry production in the region too. In addition, the toxicity of OTA has been reported to cause impairment of the gastrointestinal tract in chickens, resulting in poor nutritional absorption and hence poor growth rate [68]. Reduced BW and BW gain were observed in broiler chickens fed OTA at levels of 20 or 50 μg/kg BW [82,101] or 100 to 800 μg/kg feed [83,84], with the difference between the FCR of the group fed control and OTA-contaminated diets increasing by about 19% after 13 days of feeding in one of the studies [101]. Feeding ZEN-contaminated diets at levels of 2000 μg/kg feed was reported to reduce BW gain and increase FCR in broiler chickens [65]. Furthermore, toxicity due to T-2 (4500 or 13,500 μg/kg feed) was observed to decrease the feed intake and BW gain of broiler chickens [102], although the levels of T-2 used in the study were high and have not been reported in poultry feeds from SSA.

### 6.3. Changes in Organ Weight

Several in vitro and ex vivo studies have indicated that consumption of feeds contaminated with mycotoxins can lead to damage of various organs in chickens manifested through increased or decreased weights. In Nigeria, increased weights of the liver, spleen, and kidneys were reported in broilers fed diets contaminated with 500 μg AFB1/kg [51]. Furthermore, increased weights of the liver, heart, and kidney were observed in broilers fed AFs at EU-tolerated levels (20 μg/kg feed) [103], moderate levels (200 μg/kg feed) [71], or high levels (2000 μg/kg feed) [69]. Additionally, increased relative weights of the liver and heart were reported in broilers due to toxicity of FBs (50,000 or 200,000 μg/kg feed) [71]. Toxicity due to OTA at levels of 100 and 2500 μg/kg feed also led to increased weights of the hearts [83] and kidneys [85] of broiler chickens, respectively. In contrary reports, reduced liver weights were observed in broiler chickens fed DON at low to moderate levels (2500 to 10,000 μg/kg feed) [100] or ZEN at levels of 2000 μg/kg [65]. Deoxynivalenol toxicity has also been reported to cause damages to immune organs, such as the spleen and thymus, and alter intestinal morphology [36]. Recently, increased weights of the thymus and spleen and decreased weights of the colon were reported in broiler chickens fed DON at levels between 5000 μg/kg feed and 15,000 μg/kg feed [62,79]. Additionally, altered immune systems were reported to be linked to decreases in the size and relative weight of the spleen in broiler chickens fed T-2 (500 to 2000 μg/kg feed) [87] and decreased thymus weight in broilers fed OTA (400 or 800 μg/kg feed) [84].

### 6.4. Changes in Blood Biochemical Parameters

Changes in blood parameters are reported to be more sensitive than other parameters, such as production performance, and thus can act as indicators of mycotoxicosis in advance of other symptoms [104]. Blood parameter changes caused by impaired protein synthesis have been shown to be marked by decreased blood total protein, globulin, and albumin levels and have been linked to mycotoxicosis. Reduced plasma protein was observed in broiler chickens fed 200 μg AFB1/kg feed [104], and decreased serum total protein, albumin, and globulin were observed in broiler chickens fed high AFs levels (2000 to 5000 μg/kg feed) [72,73,75]. Toxicity due to OTA at levels of 567 μg/kg feed caused a reduction in plasma proteins, albumin, and globulins [66], and DON at levels of 10,000 μg/kg diets was associated with inhibition of hepatic metabolisms and transport, resulting in reduced serum total protein in broiler chickens [105]. However, increased serum protein was observed in broiler chickens exposed to low or moderate DON levels (1680 or 12,209 μg/kg feed) [94]. Similarly, increased total plasma protein and albumin were observed in broiler chickens due to FBs toxicity (100,000 or 200,000 μg/kg feed) [81]. Variations in the changes of biochemical parameters observed in the different studies can be attributed to age, sex, metabolic state, breed, and levels of mycotoxins and duration of exposure. Mycotoxicosis has also been associated with disruption of cholesterol biosynthesis, and in feeding trials, decreased cholesterol levels up to 24% were observed in broiler chickens fed high AFs levels (3000 to 5000 μg/kg feeds) [74,106]. In contrast, increased cholesterol levels were observed in broiler chickens fed FBs at levels of 100,000 or 400,000 μg/kg feed [81]. Disruption of renal functions due to aflatoxicosis was reported to result in decreased serum calcium levels up to 21% in broilers fed 3000 to 4000 μg AFs/kg feed [74,98] and layers fed 2500 or 5000 μg AFs/kg [52]. However, increased serum calcium levels were observed in broilers fed FBs at levels of 100,000 to 400,000 μg/kg feed [81]. Interferences with lipid metabolisms due to DON (10,000 μg/kg diet) toxicity resulted in reduced plasma triglycerides in broiler chicks [105]. In the latter study, a reduction in plasma uric acid levels was also observed. Gentles et al. [85], however, reported increased serum uric acid levels in young broilers fed OTA at levels of 2500 μg/kg. Various studies conducted in SSA indicated that OTA was not a common contaminant of poultry feed and feed ingredients, and the levels used in the latter study are considerably high and have not been reported in the field.

Mycotoxicosis causes hepatocyte degeneration and subsequent leakage of certain enzymes, such as aspartate aminotransferase (AST), alanine aminotransferase (ALT), lactic acid dehydrogenase (LDH), and gamma glutamyl transferase (GGT), into the blood [104]. However, some enzymes are synthesized not exclusively in the liver but also in the heart, kidney, brain, and skeletal muscle [70]. In experimental feeding trials, increased serum levels of AST [70,104], ALT, and GGT [70] were observed in broiler chickens fed 50 to 200 μg AFB1/kg of feed. These latter studies indicate that exposure to AFs levels such as those reported in poultry feeds in tropical regions of SSA can cause liver damages. Additionally, increases in serum ALT, LDH, and GGT levels were reported in broiler [74] and layer [52] chickens fed high AFs levels (2500 to 5000 μg/kg feed). Moreover, increases in serum AST levels in broiler chickens were linked to toxicity of FBs (200,000 μg/kg feed) [104] or ZEN (2000 μg/kg feed) [65]. Changes in creatine kinase activity has been linked to damages in tissues, and reductions in creatine kinase activities were observed in broiler chickens fed DON at levels of 5000 μg/kg feed [79]. Damaged sphingolipids due to FBs at EU-tolerated limits of 20,000 μg/kg feed were reported to cause increased serum-free Sa levels and Sa-to-So ratios in broiler chickens [59,60,61].

### 6.5. Gross or Histopathological Changes

Pathological changes such as lesions have been used as indicators of exposure to mycotoxins. Liver histopathological changes were observed in broilers fed AFB1 at EU limits of 20 μg/kg [103] or levels of 50 to 200 μg/kg diets [71,96]. Moderate to high AFs levels (750 to 5000 μg/kg feed) were also shown to cause mild to moderate liver histopathological changes in broiler [69,72,73,90,98] and layer chickens [52]. Recently, high liver and intestinal lesion scores were observed in broiler chickens fed DON (19,300 μg/kg feed) [80], and feeding FBs (50,000 or 200,000 μg/kg feed) also caused liver histological changes in broiler chickens [70]. Additionally, intestinal pathogenicity of OTA (20 or 50 μg OTA/kg body weight per day) was associated with changes in the intestinal morphology of broiler chickens characterized by lesions [82,101]. Furthermore, immunosuppressive effects of 2000 μg OTA/kg feed were linked to histopathological changes observed in the thymus and bursa of broiler chickens [67]. Chen et al. [87] reported lesions in spleens of broiler chickens fed T-2 (500 to 2000 μg/kg feed). Recently, histopathological changes were observed in renal cortical cells of broiler and layer chickens fed citrinin (CIT) at levels of 100 to 3500 μg/kg feed [40]. Toxicity due to DON (5000 to 15,000 μg/kg feed) [63,80,107] or FBs at levels recommended by EU [60] was also reported to cause intestinal damages characterized by reduced villus height and villus height and crypt depth ratio.

### 6.6. Reduced Egg Production and Egg Quality

Mycotoxicosis has been reported to be a great concern in commercial layer production, compromising egg production and egg quality [77,108]. Diets contaminated with mycotoxins are reported to cause liver malfunctioning, thus negatively affecting liver synthesis and transport of yolk precursors [78]. Reduced egg production [52] and poor egg quality [78,109] were reported in chickens fed a 2500 to 5000 μg AFs/kg diet. Furthermore, feeding AFs and DON both at levels of 2000 μg/kg feed resulted in decreased egg production and egg weight [110]. Stoev et al. [86] also observed decreased egg production and egg weight, as well as delay in the beginning of the laying period for layer hens fed OTA at levels of 1000 or 5000 μg/kg feed. Furthermore, Devegowda and Ravikiran [108] in their review reported that major mycotoxins, including AFs, ZEN, OTA, FB1, CIT, cyclopiazonic acid (CPA), and patulin, caused poor quality of eggs that are rejected as table eggs or hatching eggs, leading to significant losses in layer production.

### 6.7. Impact of Co-Occurrences of Mycotoxins on Broiler and Layer Chickens

Most toxicological studies report the toxic effects of only one mycotoxin, yet co-occurrences of mycotoxins are frequently observed in poultry feeds from SSA [8,10,33]. Enhanced toxicities due to the synergistic or additive effects of multiple contaminations compared with single mycotoxin contamination have been reported. The additive toxic effects of AFB1 (200 μg/kg feed) and FB1 (200,000 μg/kg feed) were shown to result in more pronounced toxic effects [71,104] and decreased plasma albumin, and histopathological changes in the liver and kidney were only observed in broiler chickens orally receiving 2500 μg AFB1/kg and 10,000 μg FB1/kg [111]. Chang et al. [64] reported that the combined effects of AFB1 (50 μg/kg feed) and ZEN (500 μg/kg feed) resulted in increased ZEN residues and histopathological changes in broiler chickens’ organs. Moreover, AFB1 and OTA, both at levels of 100 μg/kg feed, caused pronounced decreases in BW and OTA residues in the livers of broiler chickens [83]. Liu et al. [58] demonstrated that reduced feed intake and BW gain in broiler chickens were due to the toxic effects of DON (1500 or 5000 μg/kg feed) and FBs (20,000 μg/kg feed). Long-term feeding of diets contaminated with DON, FBs, and ZEN or DON, ZEN, and diacetoxyscirpenol below the EU regulatory limits resulted in increased FCR in broiler chickens [16]. Moreover, the combined toxic effects of DON (20,000 μg/kg feed), FBs (5000 μg/kg feed), and ZEN (500 μg/kg feed) were manifested through increased residues of ZEN and its metabolites in livers of broiler chickens [112]. Gentles et al. [85] reported the highest (35%) reduction in BW gain and pronounced changes in blood biochemicals in broiler chickens due to the combined toxicities of CPA (34,000 μg/kg feed) and OTA (2500 μg/kg feed). Furthermore, the additive toxic effects of 567 μg OTA/kg feed and 927 μg T-2/kg caused pronounced reduction in BWG and liver and kidney histological changes [66]. Reduced immune function in broiler chickens resulted from the additive immunosuppressive effects of CPA (10,000 μg/kg feed) and T-2 (1000 μg/kg feed) [93] or T-2 (500 μg/kg feed) and OTA (250 μg/kg feed) [17]. In contrary results, feeding FBs, ZEN, and DON at maximum EU-recommended values was reported to cause no toxicological interactions on broiler chickens’ productivity and health [59,113].

## 7. Postharvest Mycotoxin Mitigation Strategies in Broiler and Layer Chicken Production

**Figure 1:**
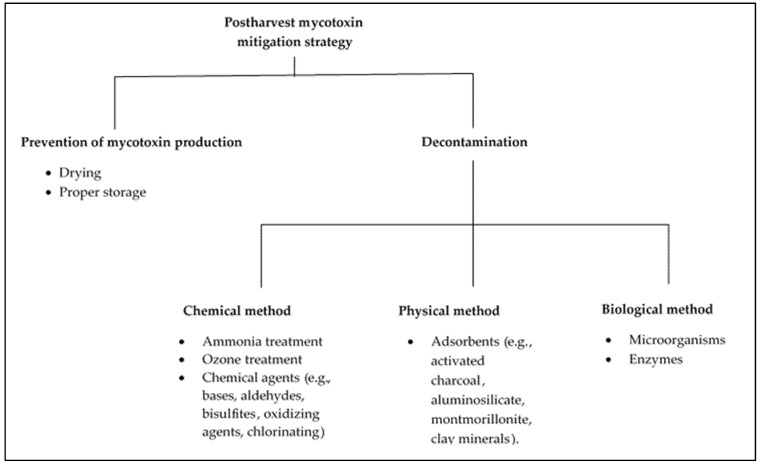
Diagrammatic representation for postharvest mycotoxin mitigation strategies in broiler and layer chickens’ production.

Figure 1 highlights techniques of preventing mycotoxin formation in crops while in the field, during transportation, and in storage. Preventing mycotoxin formation can be impractical especially with changing climatic conditions, and feasible techniques for mycotoxin decontamination, especially in feeds, have been reported to include the use of clay-based mycotoxin binders that function when the mycotoxin is already present in the diets and is being ingested by an animal [55]. They are suitable for situations where regular testing of feeds is not practical or where there is a common practice of using spoiled grains for feed formulations, such as in SSA [19,114].

Commercial clay-based mycotoxin binders are available in SSA countries, such as Nigeria [51], Kenya [114,115], and Tanzania [116], and are imported by feed processors for use in feed formulations. In most SSA countries, such as Kenya, there is no information on efficacy, safety, and regulations for use of these clay mycotoxin binders [115]. In Nigeria, commercial mycotoxin binders were shown to protect broiler chickens from the toxic effects of AFs [50,51]. In Tanzania, local clay collected from one of the regions had a good affinity for AFB1 in vitro and was relatively comparable to the commercial mycotoxin binder used in the study [116]. The use of locally available mycotoxin binders can be an economical solution for mycotoxin problems in SSA, thereby making poultry production more cost-effective; however, more trials and studies in SSA are needed to evaluate the safety and efficacy of the locally available mycotoxin binders, as the trials cited below are from outside the region.

Hydrated sodium calcium aluminosilicate (HSCAS) has a high affinity to adsorb AFB1 in vitro and prevented the negative effects of AFs in chickens in vivo [98]. Chen et al. [97], however, reported partial protection of HSCAS against the toxic effects of AFs in broiler chickens. Furthermore, HSCAS failed to completely protect broiler chickens from the toxic effects of CPA [117] and T-2 [106], indicating that the efficacy of HSCAS may be affected by the level and nature of the mycotoxin. Other clay-based compounds, including bentonite, montmorillonite, and zeolite, were also shown to ameliorate the toxic effects of AFs on chickens [72,74,75,103,109,118,119]. Clay-based compounds were reported to alleviate the toxic effects of DON in broiler chickens, although other studies have reported that these compounds are not effective against trichothecenes [105]. In contrary results, clay-based compounds, including bentonite binder, failed to completely ameliorate the toxic effects of AFs [66,69] or FBs [99]. These latter studies further demonstrate the need for an in-depth characterization of mycotoxin-binding agents for use in a given mycotoxin contamination and suitable in vivo models of target animal species to guarantee their efficacy and safety. Additionally, more research is needed to evaluate the efficiency of mycotoxin binders in the presence of more than one mycotoxin, as is common under field conditions. In experimental trials, bentonite clays were reported to protect chickens from the combined toxic effects of AFs and FBs [73] as well as AFs and OTA [83]. Additionally, another mycotoxin detoxifier consisting of a binding clay and modifying enzymes was shown to partially counteract the combined effects of OTA and T-2 toxin at levels below the CFIA-tolerated maximum limits in poultry feeds [17].

Another way of reducing the negative effects of mycotoxins already ingested and present in the gastrointestinal tract of animals involves the use of mycotoxin modifiers, such as enzymes, fungi, and bacteria, to degrade the mycotoxins into less toxic metabolites. Recently, the inclusion of *Lactobacillus* spp. in broiler chickens’ diets was shown to alleviate the toxic effects of AFB1 or AFB1 and ZEN [64] as well as DON [80]. Ma et al. [120] further demonstrated that *Bacillus subtilis* ANSB060 from fish gut ameliorated the toxic effects of AFB1 on layer chickens. Moreover, the bovine rumen bacterial strain (*Eubacterium BBSH 797*) was able to deactivate DON, forming the less toxic deepoxy-deoxynivalenol (DOM-1) [121]. Fumonisin esterase enzymes have also been shown to degrade FBs and form less toxic metabolites, including hydrolyzed fumonisin B1 (HFB1) and partially hydrolyzed fumonisin B1 (pHFB1) [122]. Inclusion of the esterase enzymes in poultry diets was reported to be safe for chickens and turkeys and efficiently degraded FBs at levels even below the EU guidance limits of 20,000 μg/kg [122]. Yeast strains have also been reported to modulate the biotransformation of OTA to less toxic OTA metabolites in ex vivo and in vivo chicken models [123]. Furthermore, *Trichosporon mycotoxinivorans*, a yeast strain from the hindgut of the termite *Mastotermes darwiniensis*, was shown to degrade OTA and ZEN to less toxic metabolites [121,124] or reduce OTA depositions in tissues [67]. Yeast cell wall extracts were also shown to have promising results in preventing the negative effects of OTA [68] and offered partial protection against the toxic effects of AFs, FBs, DON, and ZEN [95]. Microorganisms are thus suitable for the biodegradation of some mycotoxins, especially trichothecenes, which are poorly adsorbed by mycotoxin binders. However, their efficacy both practically and economically needs to be widely evaluated before commercial applications.

## 8. Conclusions

Poultry feed and feed ingredients from SSA are contaminated with mycotoxins, and co-occurrences of mycotoxins, especially AFs and FBs, are frequently observed due to colonization of feed ingredients by different mycotoxin-producing fungi. The mycotoxins have a negative impact on the health and productivity of layer and broiler chickens, resulting in significant economic losses. Additionally, chronic exposure to low levels of mycotoxins in poultry products, including eggs, meat, and liver, poses a safety concern to human consumers of these products. Few SSA countries have regulatory limits or guidance values for various mycotoxins in poultry feeds. More occurrence and toxicological information are therefore required to help monitor and control mycotoxin contamination in SSA. In addition, the safety and efficacy of local clay compounds from SSA as binding agents should be evaluated to provide suitable locally available solutions to mycotoxin problems.

## Figures and Tables

**Table 1:** Worldwide regulations of mycotoxins in poultry feeds.

Country/Region	Regulatory Limit (µg/kg)		Guidance Value (µg/kg)					Reference
	AFB1	Total AFs	DON	FBs	ZEN	T-2	OTA	
Côte d’Ivoire	-	10	-	-	-	-	-	[19]
Ghana	-	15	-	-	-	-	-	[4]
Senegal	50	-	-	-	-	-	-	[19]
South Africa	-	20	4000	50,000	-	-	20	[6]
Tanzania	5	10	-	-	-	-	-	[14]
Zimbabwe	-	10	-	-	-	-	-	[19]
EAC	20	50	-	-	-	-	-	[14]
CFIA	-	-	-	-	-	1000	2000	[17]
EU	20	-	5000	20,000	250	250	100	[16]
USA	20	-	10,000	30,000	-	-	-	[18]

Total AFs—sum of aflatoxin B1 (AFB1), aflatoxin B2 (AFB2), aflatoxin G1(AFG1), and aflatoxin G2 (AFG2); DON—deoxynivalenol; FBs—fumonisins; ZEN—zearalenone; T-2—T-2 toxin; HT-2—HT-2 toxin; OTA—ochratoxin A; CFIA—Canadian Food Inspection Agency; EAC—East African Community; EU—European Union; USA—United States of America; -means no reported regulatory limit or guidance value.

**Table 2:** Occurrences of major mycotoxins in poultry feeds and feed ingredients from SSA.

**Aflatoxins**										
**Mycotoxin**	**Country**	**Type of Sample**	**Analytical Technique**	**LOD** **(μg/kg)**	**% Positive (*n*)**	**% Above EU** **Limit**	**Max (μg/kg)**	**Mean** **(μg/kg)**	**Year of Publication**	**Reference**
AFs	Botswana	Poultry feed ingredients: peanut	TLC and HPLC–FLD	0.10	52% (29)		48.01	14.0	1998	[20]
		Poultry feeds			100% (4)		0.7	0.6		
	Cameroon	Poultry feeds: broiler feeds	Fluorimeter	2.00	93% (30)		52.0	11.1	2013	[13]
		Poultry feed ingredients: peanut meal			100% (41)	90%	950.0	161.4		
	Ethiopia	Poultry feed ingredients: maize	ELISA	1.75	88% (17)	6%	27.0		2010	[21]
		Poultry feed ingredients: groundnut	ELISA	1.75	93% (120)		11,900.0		2013	[22]
		Poultry feed ingredients: maize	LFIA	3.30	100% (150)	5%	150.0	14.7	2019	[23]
	Ghana	Poultry feeds	LFIA	3.30	100% (350)	74%	118.0	57.3	2021	[4]
	Kenya	Poultry feed ingredients	LC–MS/MS	0.10	29% (24)		99.4	38.9	2020	[10]
		Poultry feeds			93% (27)	15%	89.0	17.2		
	Malawi	Poultry feed ingredients: maize	LFIA	2.00	100% (90)	8%	140.0	8.3	2016	[24]
	Rwanda	Poultry feeds	ELISA	5.00	52% (1726)			103.8	2019	[14]
	South Africa	Poultry feeds	UHPLC–MS/MS	0.10	23% (62)		1.8	0.7	2012	[6]
		Poultry feed ingredients: maize	LC–MS/MS	0.20	10% (282)		14.0		2018	[25]
	Tanzania	Poultry feed ingredients: sunflower cakes	ELISA	1.40	80% (92)	17%	598.4		2017	[26]
		Poultry feed ingredients: sunflower seeds			59% (90)	14%	662.7			
		Poultry feed ingredients: maize and maize-based products	ELISA	2.00	32% (160)		16.2	3.4	2016	[27]
	Uganda	Poultry feeds	Fluorimeter	1.00	100% (67)	82%	393.5		2020	[11]
		Poultry feed ingredients: Maize bran			100% (4)		103.3			
AFB1	Benin	Poultry feed ingredients: peanut cake	LC–MS/MS	2.00	100% (15)		282.0		2011	[28]
	Ethiopia	Poultry feed ingredients: maize	LC–MS/MS	0.30	8% (100)		513.0	9.3	2018	[29]
		Poultry feed ingredients: maize	ELISA	1.75	34% (90)	34%	381.6		2019	[30]
	Kenya	Poultry feeds	LC–MS/MS	0.20	93% (27)	15%	38.8	10.2	2020	[10]
		Poultry feed ingredients			25% (24)		49.8	19.7		
	Nigeria	Poultry feeds	LC–MS/MS	4.00	76% (58)	62%	1067.0	198.0	2012	[31]
		Poultry feeds	LC–MS/MS		83% (30)		760.0	74.0	2018	[8]
		Poultry feed ingredients: maize			47% (17)		567.0	176.0		
		Poultry feed ingredients: peanut cake			91% (11)		3860.0	639.0		
		Poultry feed ingredients: wheat offal			30% (10)		80.0	53.0		
		Poultry feed ingredients: peanut meal	LC–MS/MS	2.00	100% (29)	90%	2820.0		2012	[32]
	South Africa	Poultry feeds	UHPLC–MS/MS	0.04	93% (105)		0.9	0.2	2019	[33]
AFB2	Kenya	Poultry feed ingredients	LC–MS/MS	0.06	17% (24)		7.0	3.4	2020	[10]
		Poultry feeds			48% (27)		4.4	1.7		
	Nigeria	Poultry feeds	LC–MS/MS	10.00	50% (58)		114.0	34.0	2012	[31]
		Poultry feeds	LC–MS/MS		50% (30)		188.0	21.0	2018	[8]
		Poultry feed ingredients: maize			24% (17)		61.0	35.0		
		Poultry feed ingredients: peanut cake			91% (11)		895.0	126.0		
	South Africa	Poultry feeds	UHPLC–MS/MS	0.02	100% (105)		7.1	0.4	2019	[33]
AFG1	Kenya	Poultry feed ingredients	LC–MS/MS	0.20	25% (24)		34.9	17.1	2020	[10]
		Poultry feeds			70% (27)		41.7	6.7		
	Nigeria	Poultry feeds	LC–MS/MS	6.00	60% (58)		235.0	45.0	2012	[31]
		Poultry feeds	LC–MS/MS		57% (30)		79.0	19.0	2018	[8]
		Poultry feed ingredients: maize			41% (17)		725.0	110.0		
		Poultry feed ingredients: peanut cake			91% (11)		568.0	157.0		
		Poultry feed ingredients: wheat offal			2% (10)		14.0	14.0		
		Poultry feed ingredients: peanut meal	LC–MS/MS	4.00	83% (29)		477.0		2012	[32]
	South Africa	Poultry feeds	UHPLC–MS/MS	0.10	97% (105)		5.2	0.7	2019	[33]
AFG2	Kenya	Poultry feed ingredients	LC–MS/MS	0.50	21% (24)		9.6	4.6	2020	[10]
		Poultry feeds			33% (27)		6.4	2.5		
	Nigeria	Poultry feeds	LC–MS/MS	10.00	10% (58)		20.0	13.0	2012	[31]
		Poultry feeds	LC–MS/MS		13% (30)		7.6	3.5	2018	[8]
		Poultry feed ingredients: maize			6% (17)		60.0			
		Poultry feed ingredients: peanut cake			55% (11)		68.0	27.0		
	South Africa	Poultry feeds	UHPLC–MS/MS	0.10	78% (105)		1.6	0.5	2019	[33]
AFM1	Kenya	Poultry feed ingredients	LC–MS/MS	0.10	21% (24)		6.9	2.9	2020	[10]
		Poultry feeds	LC–MS/MS		15% (27)		0.5	0.6		
	Nigeria	Poultry feeds	LC–MS/MS	10.00	26% (58)		29.0	15.0	2012	[31]
		Poultry feeds	LC–MS/MS		23% (30)		41.0	9.9	2018	[8]
		Poultry feed ingredients: peanut cake			73% (11)		254.0	49.0		
		Poultry feed ingredients: wheat offal			20% (10)		5.3	5.2		
		Poultry feed ingredients: maize			18% (17)		70.0	45.0		
		Poultry feed ingredients: peanut meal	LC–MS/MS	4.00	66% (29)		126.0		2012	[32]
**Type A Trichothecenes**										
**Mycotoxin**	**Country**	**Type of Sample**	**Analytical Technique**	**LOD** **(μg/kg)**	**% Positive (*n*)**		**Max (μg/kg)**	**Mean** **(μg/kg)**	**Year of Publication**	**Reference**
HT-2	Kenya	Poultry feeds	LC–MS/MS	0.50	4% (27)		13.8	13.8	2020	[10]
	South Africa		UHPLC–MS/MS	0.20	100% (105)		5.9	1.9	2019	[33]
T-2	Kenya	Poultry feeds	LC–MS/MS	0.70	4% (27)		5.2	5.2	2020	[10]
	South Africa	Poultry feeds	UHPLC–MS/MS	0.10	100% (105)		15.3	3.1	2019	[33]
		Poultry feed ingredients: maize	LC–MS/MS	2.00	1% (273)		80.0		2018	[25]
**Type B Trichothecenes**										
**Mycotoxin**	**Country**	**Type of Sample**	**Analytical Technique**	**LOD** **(μg/kg)**	**% Positive (*n*)**		**Max (μg/kg)**	**Mean** **(μg/kg)**	**Year of Publication**	**Reference**
DON	Ethiopia	Poultry feed ingredients: maize	LC–MS/MS	1.20	42% (100)		595.0	221.0	2018	[29]
		Poultry feed ingredients: maize	HPLC–FLD	30.00	29% (17)		700.0		2010	[21]
		Poultry feed ingredients: maize	LFIA	190.00	7% (150)		1980.0	650.0	2019	[23]
	Kenya	Poultry feed ingredients	LC–MS/MS	0.40	54% (27)		996.1	244.9	2020	[10]
		Poultry feeds			100% (27)		1037.0	329.1		
	Nigeria	Poultry feeds	LC–MS/MS	55.00	36% (58)		2336.0	651.0	2012	[31]
		Poultry feeds	LC–MS/MS		20% (30)		174.0	108.0	2018	[8]
		Poultry feed ingredients: wheat offal			50% (10)		837.0	578.0		
	South Africa	Poultry feed ingredients: maize	LC–MS/MS	20.00	81% (314)		9176.0		2018	[25]
		Poultry feed ingredients: wheat	HPLC–UV	50.00	82% (77)		11,022.0	943.0	2011	[34]
		Poultry feeds	UHPLC–MS/MS	2.50	99% (105)		154.0	37.8	2019	[33]
		Poultry feeds	UHPLC–MS/MS	72.00	100% (62)		1980.0	620.0	2012	[6]
**Fumonisins**										
**Mycotoxin**	**Country**	**Type of Sample**	**Analytical Technique**	**LOD** **(μg/kg)**	**% Positive (*n*)**		**Max (μg/kg)**	**Mean** **(μg/kg)**	**Year of Publication**	**Reference**
FBs	Ethiopia	Poultry feed ingredients: maize	HPLC–FLD	25.00	18% (17)		2400.0		2010	[21]
		Poultry feed ingredients: maize	LFIA	300.00	33% (150)		6520.0	680.0	2019	[23]
	Ghana	Poultry feeds	LFIA	150.00	100% (350)		15.0	1.5	2021	[4]
	Kenya	Poultry feed ingredients	LC–MS/MS	0.60	71% (24)		11,658.7	2146.2	2020	[10]
		Poultry feeds			100% (27)		2684.8	597.9		
	Malawi	Poultry feed ingredients: maize	ELISA	1000.00	84% (90)		7000.0	900.0	2016	[24]
	Rwanda	Poultry feeds	ELISA	1000.00	(1726)			1210	2019	[15]
	South Africa	Poultry feed ingredients: maize	LC–MS/MS	20.00	80% (281)		16,932.0		2018	[25]
	Tanzania	Poultry feed ingredients: maize and maize-based products	ELISA	300.00	39% (160)		62,000.0	5600.0	2016	[27]
FB1	Benin	Poultry feed ingredients: peanut cake	LC–MS/MS	1.00	7% (15)		80.0		2011	[28]
	Botswana	Poultry feed ingredients: maize	TLC and HPLC–FLD	20.00	85% (33)		1270.0	247.0	1998	[20]
		Poultry feeds			100% (4)		1050.0	572.0		
	Ethiopia	Poultry feed ingredients: maize	LC–MS/MS	3.20	70% (100)		11,831.0	606.0	2018	[29]
	Kenya	Poultry feed ingredients	LC–MS/MS	2.00	71% (24)		8345.6	1474.4	2020	[10]
		Poultry feeds			100% (27)		1926.0	431.4		
	Nigeria	Poultry feeds	LC–MS/MS	40.00	83% (58)		2733.0	964.0	2012	[31]
		Poultry feeds	LC–MS/MS		97% (30)		3760.0	1014.0	2018	[8]
		Poultry feed ingredients: maize			100% (17)		2090.0	825.0		
		Poultry feed ingredients: peanut cake			27% (11)		910.0	308.0		
		Poultry feed ingredients: wheat offal			50% (10)		67.0	37.0		
	South Africa	Poultry feeds	UHPLC–MS/MS	19.40	100% (105)		7125.0	1076.0	2019	[33]
		Poultry feeds	UHPLC–MS/MS	9.00	100% (62)		2999.0	903.0	2012	[6]
**Zearalenone**										
**Mycotoxin**	**Country**	**Type of Sample**	**Analytical Technique**	**LOD** **(μg/kg)**	**% Positive (*n*)**		**Max (μg/kg)**	**Mean** **(μg/kg)**	**Year of Publication**	**Reference**
ZEN	Botswana	Poultry feed ingredients: peanut	TLC and HPLC–FLD	20.00	5% (20)		40.0	40.0	1998	[20]
		Poultry feeds			25% (4)		40.0	40.0		
	Ethiopia	Poultry feed ingredients: maize	LC–MS/MS	0.12	96% (100)		1656.0	92.0	2018	[29]
	Kenya	Poultry feed ingredients	LC–MS/MS	0.20	83% (24)		910.4	71.3	2020	[10]
		Poultry feeds			100% (27)		873.4	103.4		
	Nigeria	poultry feeds	LC–MS/MS		83% (30)		71.0	9.3	2018	[8]
		Poultry feed ingredients: wheat offal			90% (10)		67.0	19.0		
		Poultry feed ingredients: peanut cake			18% (11)		1.1	0.9		
		Poultry feed ingredients: maize			65% (17)		4.8	1.2		
	South Africa	Poultry feeds	UHPLC–MS/MS	0.10	100% (105)		428.9	71.2	2019	[33]
		Poultry feeds	UHPLC–MS/MS	3.50	100% (62)		610.0	100.0	2012	[6]
		Poultry feed ingredients: maize	LC–MS/MS	4.00	47% (308)		6276.0		2018	[25]
**Ochratoxin A**										
**Mycotoxin**	**Country**	**Type of Sample**	**Analytical Technique**	**LOD** **(μg/kg)**	**% Positive (*n*)**		**Max (μg/kg)**	**Mean** **(μg/kg)**	**Year of Publication**	**Reference**
OTA	Benin	Poultry feed ingredients: peanut cake	LC–MS/MS	0.10	33% (15)		2.0		2011	[28]
	Ethiopia	Poultry feed ingredients: maize	ELISA	1.90	24% (150)		186.5	8.2	2019	[23]
	Kenya	Poultry feeds	LC–MS/MS	1.00	19% (27)		10.6	4.8	2020	[10]
		Poultry feed ingredients			8% (24)		1.1	0.6		
	Nigeria	Poultry feeds	LC–MS/MS	4.00	34% (58)		26.0	10.0	2012	[31]
		Poultry feeds	LC–MS/MS		27% (30)		15.0	5.4	2018	[8]
		Poultry feed ingredients: maize	LC–MS/MS		12% (11)		3.1	2.2		
		Poultry feed ingredients: peanut cake			55% (11)		127.0	35.0		
	South Africa	Poultry feed ingredients: maize	LC–MS/MS	0.20	7% (269)		95.0		2018	[25]

AFs—total aflatoxins (AFB1 + AFB2 + AFG1 + AFG2), AFB1—aflatoxin B1, AFB2—aflatoxin B2, AFG1—aflatoxin G1, AFG2—aflatoxin G2, AFM1—aflatoxin M1, FBs—fumonisins, FB1—fumonisin B1, DON—deoxynivalenol, ZEN—zearalenone, T-2—T-2 toxin, HT-2—HT-2 toxin, OTA—ochratoxin A, LOD—limit of detection, ELISA—enzyme-linked immunosorbent assay, TLC—thin-layer chromatography, HPLC–FLD—high-performance liquid chromatography with fluorescence detection, UHPLC–MS/MS—ultra-high-performance liquid chromatography tandem mass spectrometry, LC–MS/MS—liquid chromatography tandem mass spectrometry, LFIA—lateral flow immunochromatographic assay, *n*—number of samples, EU regulatory limit for AFB1—20 μg/kg, mean—mean concentration of positives.

**Table 3:** Effects of major mycotoxins on layer and broiler chickens’ health and productivity and presence of residues.

Mycotoxin	Dosage (mg/kg Diet)	Species	Age at Start of the Trial (Days)	Period of Exposure (Days)	Effects Observed	Reference
AFs	0.02	Broilers	1	35	Increased liver and kidney weights Decreased serum albumin, ALP, and ALT	[69]
AFB1	0.05	Broilers	3	42	Decreased BW gain and FI Decreased serum g-GGT, AST, and ALT Residues of AFB1 and AFM1 in livers	[70]
	0.1	Broilers	3	42	Decreased BW gain and FI Decreased serum g-GGT, AST, and ALT Residues of AFB1 and AFM1 in livers and muscle	
	0.2	Broilers	8	33	Decreased BW and BW gain Decreased mean antibody titers against vaccine for Newcastle disease Hepatic histopathology changes	[71]
	0.5	Broilers	1	56	Decreased BW and BW gain Increased FCR Increased mortality Increased liver, spleen, and kidney weights	[51]
	2	Broilers	1	21	Decreased BW gain and FI Decreased serum Prot, Alb, Ca, and Glu Increased liver weights Hepatic histopathology changes	[72]
	2.5	Broilers	23	27	Decreased BW gain Decreased serum Prot, Alb, and Glob Increased liver weights Hepatic histopathology changes	[73]
AFs	3	Broilers	1	42	Decreased BW and BW gain Decreased serum Prot, Ca, K, and Chol Increased liver weights	[74]
AFB1	5	Broilers	30	22	Decreased BW gain Decreased serum Prot, Alb, and Glob Increased liver weights Hepatic histopathology changes	[75]
AFs	5	Broilers	1	21	Decreased BW gain and FI Decreased hepatic vitamin A levels Increased liver weights	[76]
	0.05	Layers	210	60	Decreased FI Residues of AFB1 in eggs	[77]
	0.10	Layers	210	60	Decreased FI Residues of AFB1 in eggs	
AFB1	2.5	Layers	308	28	Decreased egg quality Residues of AFB1 in livers	[78]
AFs	5	Layers	189	32	Decreased egg production Decreased serum trig, Ca, P, AST, and ALT Increased liver weights Hepatic histopathology changes	[52]
DON	15	Broilers	1	42	Decreased BW gain Increased FCR Increased weight of thymus and gizzard Decreased weight of colon Decreased cholesterol Changes in small intestine morphometry	[79]
	19.3	Broilers	6	8	Decreased villi height Increased crypt depth Decreased intestinal health	[80]
FB1	20	Broiler	1	35	Increased Sa/So and Sa	[59]
	50	Broilers	8	33	Decreased BW and BW gain Decreased mean antibody titers for vaccine against Newcastle disease Hepatic histopathology changes	[71]
	100	Broiler	1	28	Decreased FI and BW Increased FCR Increased liver weights Increased Sa/So Increased serum Prot, Alb, Chol, Trig, Ca, ALT, and AST Decreased villus height and villus-to-crypt ratioHepatic histopathology changes	[81]
	200	Broilers	8	33	Decreased BW and BW gain Decreased mean antibody titers against vaccine for Newcastle disease Increased liver weights Hepatic histopathology changes	[71]
ZEN	2	Broilers	1	42	Decreased BW gain Increased FCR Increased liver weight Increased serum AST and ALT levels Residues of ZEN in liver and kidney	[65]
OTA	0.05	Broilers	7	28	Decreased BW gain Decreased leukocyte and lymphocyte count Intestinal mucosa architecture alterations	[82]
	0.1	Broilers	1	42	Decreased BW Increased heart weight Residues of OTA in liver	[83]
	0.4 or 0.8	Broilers	1	35	Decreased BW and FI Decreased thyroxine concentration Decreased WBC, humoral immune response, and cell-mediated immunity Increased gizzard weight Increased mortality Anemia	[84]
	2.5	Broilers	1	21	Decreased BW gain Decreased serum Prot, Alb, and Chol Increased serum uric acid and Trig Increased weight of kidney	[85]
	5	Layers	14	365	Decreased egg weights Decreased egg production Delay of the beginning of the laying period	[86]
T-2	2	Broilers	1	21	Decreased spleen weight and size Decreased CD4+/CD8+ Increased apoptotic splenocytes Lesions in spleen	[87]

AFs—sum of aflatoxin B1, aflatoxin B2, aflatoxin G1, and aflatoxin G2; AFB1—aflatoxin B1; AFM1—aflatoxin M1; FB1—fumonisin B1; DON—deoxynivalenol; ZEN—zearalenone; T-2—T-2 toxin; OTA—ochratoxin A; BW—body weight; FI—feed intake; FCR—feed conversion ratio; Prot—protein; Alb—albumin; Glob—globulin; Glu—glucose; Chol—cholesterol; Trig—triglyceride; Ca—calcium; K—potassium; P—phosphorus; WBC—white blood cells; Sa—sphinganine; Sa/So—sphinganine-to-sphingosine ratio; AST—aspartate aminotransferase; ALT—alanine aminotransferase; ALP—alkaline phosphatase; g-GGT—gamma glutamyl transferase.
